# dbfishR: An R package for fish and water quality data access and entry

**DOI:** 10.1016/j.dib.2026.113118

**Published:** 2026-07-30

**Authors:** Matthew J. Wilson, Sara A. Ashcraft, Mark A. Kirk, Daniel E. Ressler

**Affiliations:** a Susquehanna University, 514 University Ave., Selinsgrove PA, 17870, United States; b Allegheny College, 520N Main St, Meadville, PA 16335, United States

**Keywords:** Pennsylvania, Stream ecology, Microsoft Access, Biodiversity, Community ecology

## Abstract

The dbfishR package is designed to access data collected as part of Pennsylvania’s Unassessed Waters Initiative, a state-wide program to sample fish communities and water quality in headwater streams throughout the state, currently including 1491 stream sites collected between 2010–2025. With the package database, we have also released a robust database template to both facilitate collaboration and future data contributions through data standardization for in-stream collection of data related to water quality; discharge; site metadata, and fish counts, lengths, and weights. The database includes structured forms with value limits for data entry to support efficiency, accessibility, and quality control. The package is structured for tidy import from the relational database structure for ease of data analysis and comparison with external data. Because of these data and query structures, the package and database can be expanded to include data from any aquatic research involving substrate, water quality, or fish and amphibians. We present the package and database as readily available access to the existing dataset while offering opportunity for future contribution, specifically from the eastern United States, as well as adaptability for use in data curation and access for watersheds globally.

Specifications Table

**Table:** 

Subject	Earth & Environmental Sciences
Specific subject area	R package for public use of stream biotic (fish and future incorporation of amphibians) and abiotic (water quality and hydrological variables) data
Type of data	Table (R data frame from package functions) Relational database (Microsoft Access) Supporting materials (functions, code vignette, text readme)
Data collection	Data were collected from 1491 stream reaches between 2010–2025 during the May-August window via backpack electrofishing of 100 m stream reaches (Smith-Root LR-24 units) for fish, then weighed with OHAUS Scout balances to 0.1 g and total lengths to nearest mm via measuring boards. Water quality was sampled with HANNA Instruments multimeter probes for dissolved oxygen, pH, temperature, and conductivity. Turbidity was measured with a LaMotte turbidimeter. Hardness was measured with a Hach total hardness field kit. Stream reach wetted width was measured at five locations with a visual assessment of flow conditions and discharge was measured with a Marsh McBirney flowmeter for velocity and a USGS top setting wading rod for depth profile at a subset of locations. Site metadata include observational notes, site latitude and longitude, and weather conditions during sampling.
Data source location	City/Town/Region: Pennsylvania Country: United States
Data accessibility	Repository name: Zenodo Data identification number: https://doi.org/10.5281/zenodo.20074383 Direct URL to data: https://zenodo.org/records/20074383 Instructions for accessing these data: https://github.com/Team-FRI/dbfishR
Related research article	none

## 1 Value of the Data

• These data are valuable for understanding headwater fish species occurrence, demographics, and relationship to water quality and stream size.
• Researchers, natural resource managers, state agencies, and local watershed organizations can use these data to identify local and regional patterns for decision making or ecological questions.
• This dataset represents 1491 unique stream sites across Pennsylvania with 15,112 fish records of length and weight.
• The database structure is designed for flexibility to be repurposed or incorporate additional contributions to improve the spatial scope and resolution of available data over time.

## 2 Background

Across watershed ecology, management, and conservation there are significant gaps between the parties involved in long-term research, monitoring, and policy for data use and access, despite the benefits of connecting these groups [1]. From these diverse drivers of data collection it can be difficult to translate data from one initial purpose to another, such as management to research [2]. In addressing these challenges, our package allows ease of access to a large stream dataset, provides a standardized database template that serves as a flexible yet robust foundation to adapt for other ecological data collection, and it allows for ease of data entry and incorporation into the dbfishR package for future releases. While there has been a rapid growth in research surrounding citizen science data collection and collaboration [3,4], there has been limited focus on making professionally collected data available by comparison, particularly in aquatic ecosystems. Importantly, most R packages that support fish and fisheries related data accessibility, such as FSA [5] and TropFishR [6], are designed for economically important analyses such as stock assessments rather than basic ecological research analyses. The database and functions in dbfishR take an ecological approach to aquatic data that include fish sampling, enabling more broad applications.

## 3 Data Description

This article describes stream fish data collected as part of Pennsylvania’s Unassessed Waters Initiative and augmented by additional water quality, discharge, and site data to allow for broader ecological comparisons. These surveys were conducted between 2010 and 2025 across the state of Pennsylvania in small wadeable streams (defined as less than chest deep and 12.5 m wide [7]), primarily 1st and 2nd Strahler Order headwater streams [8], that had not previously been sampled by a state agency for fish (mean ± SD of average wetted width 2.2 ± 1.6 m). Based on the widest and narrowest streams with flowing water at the time of sampling, upstream watersheds ranged in size from 0.32 km^2^ to 51.22 km^2^ and were up to 4th Order in size. Streams were only resampled in multiple years or at multiple sites if >0 and <3 of one species of trout were sampled in a 100 m reach. This is because multiple life stages (estimated by size) and 3 or more individuals (or all young-of-year) are required to designate a stream for natural trout reproduction in Pennsylvania. In total, this dataset includes samples from 1491 unique stream sites and 15,112 records of fish lengths and weights. Notably, these records also include sampling events where no fish were found and when no sampling occurred due to the absence of surface water in the channel.

All data are contained in the “Complete Data” release of the dbfishR package in the “DataSource” Microsoft Access database [9]. These data can either be accessed via installation of the R package and dbfishR functions or manual download of the Access database. Both options pull from the same schema, which is summarized in Table 1. All tables contain fields for “SiteCode” (for time-independent fields and tables) and/or “EventCode” (for time-dependent fields and tables) to allow all fields to be merged by matching sampling events or site data. Note, as amphibians are commonly captured as bycatch in electrofishing surveys and measures of their species, length, and weight could easily follow the same format as fish sampling, we include a table and structure for incorporation of these data into future releases of “Complete Data” to allow for more broad utility of the package and database.

**Table 1:** Data schema and naming convention between dbfishR and “Complete Data” MS Access database.

dbfishR Function	MS Access Table Returned	Data Description
get_discharge_event_info	DischargeEventInfo	Stream width (m), depth (m), and recorder metadata for discharge.
get_discharge_records	DischargeRecords	Individual stream velocity measures (m/s) used to calculate stream discharge.
get_events	Events	Sampling event metadata and water quality samples.
get_fish_records	FishRecords	Individual fish length (mm), weight (g), and health condition (deformity, erosion, lesion, tumor, or parasite).
get_fish_survey	FishSurvey	Electrofishing sampling information, including sample effort, stream wetted width, visual assessment of flow, and metadata.
get_fish_tally	FishTally	Number of fish sampled during each survey by species.
get_sites	Sites	Site metadata, for linking sampling events over time, and access metadata for public lands.

## 4 Experimental Design, Materials and Methods

### 4.1 Site selection

Prioritized waters were determined by Pennsylvania Fish and Boat Commission’s Unassessed Waters Initiative staff and most are small headwater streams (53% under 2 m, 34% 2–4 m, and only 3% over 6 m in wetted width). Surveyors received the latitude and longitude of the targeted stream’s confluence and the headwaters. Surveys were conducted within 0.8 km from the stream’s confluence with the site chosen based on habitat, i.e., surface water, connectivity with the adjacent waters, etc. All sampling was completed between May 20th and October 1st of the sampling year during low- or normal-flow conditions, avoiding high-flow conditions after precipitation events. Detailed site location descriptions with latitude and longitude were collected.

### 4.2 Fish sampling

We collected fish via backpack electrofishing of 100 m stream reaches (Smith-Root LR-24 units) for fish, then weighed with OHAUS Scout balances to 0.1 g and measured total lengths to nearest mm. Sampling method and resolution of length and weight contained in the database varies by year and partner organization. In the first year of the project (2010), triple-pass depletion of a 100 m reach was completed (electrofishing of the same reach three times in sequence), and in subsequent years the standard requirement was single-pass, extending past 100 m if needed to meet designation requirements for natural trout reproductive stream. Even though fish were weighed and total length measured, only length categories of 25 mm were required to be recorded by the Fish and Boat Commission (0–25 mm, 26–50 mm, 51–75 mm, etc.). As a result, some sampling events include individuals in binned categories and others include the fine resolution measured in the field. Similarly, nongame species were enumerated in abundance categories as rare (1 individual per 100 m reach), present (2–8), common (9–33), and abundant (>33), following PA Fish and Boat Commission categories. All fish were immediately released into the reach where they were collected following measurement.

### 4.3 Water quality and stream size

We sampled water quality with multimeter probes for dissolved oxygen, pH, temperature, and conductivity (e.g., HANNA Instruments, YSI, and OBrien brands), as well as alkalinity and hardness using Hach field kits and turbidity with a LaMotte turbidimeter. We collected all water samples in the mid-depth of the thalweg within the sampled reach. In addition, all stream reaches were categorized based on their size. At five locations along the sampled reach (0, 25, 50, 75, and 100 m) we measured wetted stream width and, for a subset of reaches, calculated stream discharge using 10 equidistant measures of velocity at 60% of water depth across the channel with a Marsh McBirney flowmeter and top setting wading rod at the location having the simplest channel cross-section and within 10 m of the downstream end of the reach. Within all reaches we visually assessed the streamflow conditions based on Fish and Boat category designations of dry, low, normal, and high.

### 4.4 Data entry and curation

We transcribed all data from field data sheets to a blank copy of the Microsoft Access database included in the dbfishR package. After data entry, quality assurance measures included plotting fish length/weight (if applicable) to identify and check outliers, searches for missing data (i.e., blank entries), and comparisons of water quality values known to be correlated (e.g., water temperature and dissolved oxygen) to identify any additional outliers. After this initial round of checks, we used a random check of at least 1% of digital and written values to confirm matches prior to appending data to the master database. Future contributions to the database will be vetted by the authors following this approach and all data that meet quality standards will be incorporated into future releases.

## Limitations

There are two limitations to use for fish records from these data. One is that all species other than *Salvelinus fontinalis, Salmo trutta*, and *Oncorhynchus mykiss* were not consistently measured for length and weight. Instead, other species were given designations of “Rare”, “Present”, “Common”, and “Abundant” which are based on Pennsylvania Fish and Boat designations that were not consistently applied across observers or over time. The other limitation is that some records of length do not have associated weights and/or are binned by 25 mm length ranges, to match original reporting requirements. In addition, though all sites include wetted width and a visual assessment of flow, there are limited discharge and velocity measurements associated with these data to better quantify stream hydrologic conditions.

## Ethics Statement

The authors have read and follow the ethical requirements for publication in Data in Brief and confirm that the current work does not involve human subjects, animal experiments, or any data collected from social media platforms. All field sampling was completed under Pennsylvania Fish and Boat Commission permits and appropriate university IACUC approval.

## CRediT Author Statement

**Matthew Wilson**: Conceptualization, Methods, Software, Validation, Investigation, Formal analysis, Resources, Data Curation, Writing - Original Draft, Writing - Review & Editing, Funding acquisition. **Sara Ashcraft**: Methods, Validation, Investigation, Formal analysis, Resources, Data Curation, Writing - Review & Editing, Funding acquisition. **Mark Kirk**: Validation, Investigation, Resources, Data Curation, Writing - Review & Editing, Funding acquisition. **Daniel Ressler**: Conceptualization, Methods, Software, Validation, Investigation, Formal analysis, Resources, Data Curation, Writing - Review & Editing.

## Animal Subject

This study was conducted in accordance with the ARRIVE (Animal Research: Reporting of In Vivo Experiments) guidelines as well as the following guidelines for animal welfare and/or reporting: Institutional Care and Use Committee (CITI guidelines).

This study was approved by IACUC at Susquehanna University.

## Data Availability

GitHubdbfishR (Original data)
